# Burden of Hospitalization for Coccidioidal Meningitis: An Analysis of the National Inpatient Sample, 2019–2021

**DOI:** 10.1093/ofid/ofae706

**Published:** 2024-11-27

**Authors:** Craig I Coleman, Fariba Donovan, Lahar Miriyapalli, Ryan Shan, Belinda Lovelace

**Affiliations:** School of Pharmacy, University of Connecticut, Storrs, Connecticut, USA; Valley Fever Center for Excellence, University of Arizona College of Medicine–Tucson, Tucson, Arizona, USA; Division of Infectious Diseases, Department of Medicine, University of Arizona College of Medicine–Tucson, Tucson, Arizona, USA; BIO5 Institute, University of Arizona, Tucson, Arizona, USA; School of Pharmacy, University of Connecticut, Storrs, Connecticut, USA; School of Pharmacy, University of Connecticut, Storrs, Connecticut, USA; Health Economics and Outcomes Research, F2G Inc, Princeton, New Jersey, USA

**Keywords:** burden of illness, coccidioidomycosis, epidemiology, meningitis, prevalence

## Abstract

Coccidioidal meningitis (CM) requires lifelong aggressive management, often necessitating hospitalization. In the National Inpatient Sample (2019–2021), CM hospitalizations (N = 2305) were associated with frequent CM-related procedures (63.6% [95% confidence interval {CI}, 59.3%–67.6%]), long stays (mean, 13.0 days [95% CI, 11.3–14.6 days]), substantial costs per stay (mean, $48 155 [95% CI, $42 382–$53 929]), and a 7.6% (95% CI, 5.6%–10.3%) mortality incidence. CM inflicts a substantial burden on hospital resources.

## Teaser

Coccidioidal meningitis (CM) often necessitates hospitalization. CM hospitalizations are associated with frequent CM-related procedures, protracted hospital stays with substantial costs and a case fatality rate of nearly 8%. CM inflicts a substantial burden on hospital resources.

Coccidioidomycosis is a fungal infection endemic to the southwestern United States (US), as well as parts of Mexico and Central and South America [1]. Coccidioidal meningitis (CM) is the most severe and fatal form of coccidioidomycosis. It is associated with considerable morbidity and mortality due to associated central nervous system (CNS) complications, the need for devices to treat these complications, and adverse events related to high-intensity, lifelong antifungal therapy [1–3].

Estimates of the burden of hospitalization for coccidioidomycosis have been previously reported [4, 5]. However, there is a paucity of data evaluating the burden of hospitalization in patients with CM specifically. In this study, we sought to assess the hospital burden associated with CM, including all-cause inpatient mortality, healthcare utilization, and all-cause total hospital costs.

## METHODS

This retrospective, observational cohort study was performed using the National Inpatient Sample (NIS), Healthcare Cost and Utilization Project (HCUP), Agency for Healthcare Research and Quality, from 2019 to 2021 [6]. The NIS is a 20% stratified systematic randomized sampling of all hospital discharges, drawn from states participating in HCUP (including all states in which coccidioidomycosis is considered endemic) and covering more than 97% of the US population with all payer types. NIS data are compiled using a complex survey design, requiring the use of survey-specific analysis methods that simultaneously account for clustering and stratification and the potential for sampling bias. Doing so allows weighting of estimates to generate nationally representative estimates with accompanying variance estimates [7].

Inclusion criteria for this study consisted of hospitalizations in adults (≥18 years of age) with a diagnosis of CM. CM was identified by an *International Classification of Diseases, 10th Revision* (*ICD-10*) diagnosis code of B38.4 in a primary or nonprimary coding position. We performed descriptive analysis on the weighted (nationally representative) set of CM hospitalizations and report frequencies, mean with 95% confidence intervals (CIs), or median with range values for demographics, comorbidities, and outcomes of interest. Outcomes included the need for CM-related procedures (ie, ventricular shunt, drain, or infusion device placement, revision, or removal), hospital length of stay (LOS), all-cause total hospital treatment costs (in 2023 US dollars [US$]), and all-cause inpatient mortality. Charges were converted to costs using HCUP-supplied cost-to-charge ratios. Costs were inflated to 2023 US$ based upon the Consumer Price Index for Medical Care obtained from the Bureau of Labor Statistics [8]. Database management and statistical analysis were performed using IBM SPSS Statistics software, version 28.0.1.1 (IBM, Armonk, New York).

Use of the NIS data was determined to be exempt from institutional review board oversight, as this study did not involve human subjects research, and the investigators were supplied only de-identified and Health Insurance Portability and Accountability Act–compliant data.

## RESULTS

In 2019–2021, there were 101 108 570 weighted hospitalizations in the NIS. Of these, 28 915 had an *ICD-10* diagnosis code for any type of coccidioidomycosis, and a subset of 2305 (8.0%) had a diagnosis code for a CM hospitalization in adult patients. This equated to annual rates of hospitalizations for CM ranging from 2.7 to 3.2 cases per million Americans.

Table 1 presents demographic, clinical, and hospital-related characteristics of patient discharges with CM. The mean age was 47.9 years (95% CI, 46.3–49.5 years; median, 47). Hospitalizations predominantly occurred among men (73.3% [95% CI, 68.8%–77.4%]) and occurred primarily in the West census region (89.7% [95% CI, 88.6%–90.8%]). Combined, more than two-thirds of the hospitalizations were among White (31.5% [95% CI, 27%–36.4%]) or Hispanic (37.2% [95% CI, 37.2%–42.0%]) patients. Hospitalizations with CM were similarly distributed across the 3 years of NIS data utilized.

**Table 1.** Demographics, Comorbidities, and Outcomes of Hospitalizations for Coccidioidal Meningitis

Characteristics	Coccidioidal Meningitis (N = 2305 Hospitalizations)
Demographics	
Age, years	
Mean (95% CI)	47.9 (46.3–49.5)
Median (range)	47 (18–90)
Sex	
Female	26.7 (22.6–31.2)
Male	73.3 (68.8–77.4)
Race/ethnicity	
White	31.5 (27–36.4)
Black	14.2 (11.1–18.1)
Hispanic	37.2 (37.2–42.0)
Asian or Pacific Islander	10.9 (8.1–14.6)
Other	4.8 (4.2–5.6)
Hospital region	
West	89.7 (88.6–90.8)
Other	10.3 (9.2–11.4)
Primary payer	
Medicare	26.9 (22.6–31.7)
Medicaid	39.5 (34.7–44.4)
Commercial/private	25.8 (21.7–30.4)
Other	6.3 (5.5–7.2)
Year	
2019	35.8 (27.9–44.5)
2020	29.9 (23.0–37.9)
2021	34.3 (26.8–42.6)
Comorbidities	
Diabetes	40.2 (38.8–41.5)
Smoking	36.8 (35.0–38.7)
Sepsis	17.4 (14.2–21.0)
Immunocompromised^a^	17.1 (14.2–20.6)
HIV	8.7 (6.6–11.4)
Cancer	2.8 (1.7–4.8)
Autoimmune diseases	3.5 (2.0–5.9)
Other immunocompromised states	3.3 (1.9–5.6)
Drug abuse	9.5 (7.3–12.4)
COPD or asthma	9.3 (6.9–12.5)
Obesity	8.0 (5.8–11.0)
Alcohol abuse	3.7 (2.4–5.7)
Cirrhosis	3.3 (2.0–5.2)
End-stage renal disease	2.4 (1.3–4.2)
Coccidioidomycosis presentation	
Pulmonary coccidioidomycosis	18.2 (14.7–22.3)
Meningitis complication^b^	22.8 (18.9–27.2)

Data are presented as % (95% confidence interval) unless otherwise indicated.

Abbreviations: CI, confidence interval; COPD, chronic obstructive pulmonary disease; HIV, human immunodeficiency virus.

^a^Includes HIV, cancer, autoimmune diseases, myelodysplastic syndrome, neutropenia, transplant, and other immunosuppressive disorders (*International Classification of Diseases, Tenth Revision* codes D80–D89). Not all subtypes of immunocompromised states are reported due to the Healthcare Cost and Utilization Project's reporting “Rule of 10.”

^b^Primary diagnosis code for a common meningitis complication including hydrocephalus, cerebrovascular accident, encephalitis, encephalopathy, cerebral vasculitis or vasospasm, cerebral abscess, shunt complication, or infusion device failure.

Table 2 depicts outcomes of patient discharges with CM. More than one-fifth (22.8% [95% CI, 18.9%–27.2%]) of hospitalizations had a primary billing code for a meningitis complication. Concurrent pulmonary coccidioidomycosis was present in 18.2% (95% CI, 14.7%–22.3%) of patient hospitalizations, and 17.1% (95% CI, 14.2%–20.6%) of hospitalizations were considered to have occurred in immunocompromised patients. At least 1 CM-related procedure was required during 63.6% (95% CI, 59.3%–67.6%) of hospital stays. Mean LOS was 13.0 days (95% CI, 11.3–14.6 days; median, 7 days), and mean all-cause total treatment costs were $48 155 (95% CI, $42 382–$53 929; median, $25 564) per stay. In total, 7.6% (95% CI, 5.6%–10.3%) of all hospitalizations resulted in death prior to discharge.

**Table 2.** Outcomes of Hospitalizations for Coccidioidal Meningitis

Outcomes	Coccidioidal Meningitis (N = 2305 Hospitalizations)
CM-related procedure^a^, % (95% CI)	63.6 (59.3–67.6)
Died in hospital, % (95% CI)	7.6 (5.6–10.3)
Length of stay, days	
Mean (95% CI)	13.0 (11.3–14.6)
Median (range)	7 (0–198)
Hospital costs, $ ×1000	
Mean (95% CI)	48.2 (42.4–53.9)
Median (range)	25.6 (1.1–541.3)

Abbreviations: CI, confidence interval; CM, coccidioidal meningitis.

^a^Includes ventricular shunt, drain, or infusion device placement, revision, or removal.

Subgroup analysis stratified by whether or not the hospitalization included a CM-related procedure showed that the need for a procedure was associated with a longer LOS (Δ = 4.0 days [95% CI, .82–7.2 days]) and increased all-cause total hospital costs (Δ = $24 875 [95% CI, $13 766–$35 984]). No difference in all-cause inpatient mortality was observed (odds ratio, 0.66 [95% CI, .33–1.30]).

## DISCUSSION

Our study included a large cohort (N = 2305) of hospitalizations for patients with CM. The mean age was approximately 48 years, the proportion of hospitalizations occurring in men was about three-quarters, the West region was the location of nearly 90% of hospitalizations, and hospitalizations occurred in White or Hispanic patients about two-thirds of the time. These demographics were consistent with prior studies of all types of coccidioidomycosis diagnoses [1–4, 9].

We observed substantial all-cause hospital treatment costs associated with CM (mean, $48 155; median, $25 564). In a prior study by Chu and colleagues [5] of an all-coccidioidomycosis (both CM and non-CM) population, median hospital charges were approximately $25 700. While these values appear similar, it is important to note that costs and charges are not the same. Charges typically reflect a markup for profit, whereas costs represent the total monetary expenses for providing care. In our study we obtained costs of providing care by converting charges to costs using HCUP-supplied cost-to-charge ratios [6]. Consequently, our data likely suggest that the cost of a hospitalization for a CM is greater than that of an all-comer coccidioidomycosis admission. Unlike prior NIS analyses of all coccidioidomycosis diagnoses [4, 5], our CM analysis leveraged the detailed procedural billing code data provided in the NIS to assess whether CM-related procedures occurred during the hospitalization. About 64% of hospitalizations for CM in our analysis had a procedure code for ventricular shunt, drain, or infusion device placement, revision, or removal. This finding is consistent with the Infectious Diseases Society of America guidelines [2] that suggest surgical intervention is often needed (and repeated) to treat CM in addition to antifungal therapy, which is lifelong and often difficult to adhere to or requires frequent regimen modifications. Moreover, Sivasubramanian and colleagues [9], who followed 133 CM patients from the time of diagnosis through a median of 36 months (interquartile range, 8–72 months), found that 72% of patients required surgical intervention for the management of CM and 53% of ventricular shunts required revision over the course of study follow-up. Due to the frequency and expense associated with surgical procedures to treat CM, we believe it is reasonable to hypothesize the average CM hospitalization would be longer and more expensive than the average coccidioidomycosis stay. To support this hypothesis, we performed a subgroup analysis stratified by whether the CM hospitalization included a related CNS procedure or not. Among those needing a CM-related procedure, the average LOS was 4 days longer and had an incrementally greater average total cost of $24 875 more than those not receiving a CNS procedure.

Not surprisingly, our 7.6% incidence of death for CM hospitalizations appeared to be higher than estimates seen in prior NIS analyses of all adult coccidioidomycosis hospitalizations [1–4]. Chu et al [5] observed a 6% incidence of inpatient mortality using 2002 NIS data. Luo et al [4], using 2005–2012 NIS data, reported an overall mortality incidence of 2.7% for all coccidioidomycosis hospitalizations (ranging from 1.1% to 5.2% across individual years). The proportion of CM patients in our cohort versus prior NIS cohorts may explain observed differences in mortality. Of note, Sivasubramanian et al [9], in a study not performed in a NIS dataset and having an extended follow-up, reported an all-cause mortality incidence of near 22% over 36 months of follow-up in which CM patients were hospitalized on average 3 times per patient (range, 1–9).

Our study has several limitations. Like any retrospective database study, misclassification due to miscoding may have been present and cannot be ruled out [10]. No validation studies assessing the sensitivity and specificity of coccidioidomycosis billing codes have been published, but it is likely that the *ICD-10* coding used had higher specificity and lower sensitivity, hence missing cases of CM. Consistent with prior studies [3, 4], we defined CM as an appropriate diagnosis code present in any coding position. This allowed us to limit missed CM hospitalizations in NIS but may have resulted in the inclusion of patients admitted with, and not for, CM. NIS data only provide prevalence data on the hospitalizations for CM and do not allow us to identify how long they have had CM. The cause of mortality is not distinguishable in NIS and only reports data through hospital discharge. Consequently, we report on all-cause inpatient mortality only. NIS does not contain medication use or outpatient data; therefore, we cannot comment on the usage, treatment patterns, or appropriateness of antifungal treatment in our cohort or subsequent healthcare resource utilization.

## CONCLUSIONS

This study is a novel investigation of the hospital burden of CM in adult patients. CM inflicts a substantial burden on hospital resources. Further studies of the costs and consequences of CM are needed using large datasets, with more detailed medication utilization, clinical, and laboratory data that allow for more thorough assessment of antifungal use patterns and outcomes over longer follow-up periods.

## Notes

***Financial support***. This work was supported by F2G, Inc., Princeton, New Jersey.
